# Umbilical cord mesenchymal stem cell‐derived exosomes promote axon regeneration during optic nerve injury through microRNA‐dependent mTORC1 signalling

**DOI:** 10.1002/ctm2.1319

**Published:** 2023-07-03

**Authors:** Xuan Sang, Lei Tang, Liang Zhao, Nana Xu, Feng Liu, Yuhui Shen, Wei Wei, Yaqi Cheng, Wanjing Huang, Yurun Liu, Yaru Su, Chao Xu, Yongsheng Li, Zhichong Wang, Sheng Liu

**Affiliations:** ^1^ State Key Laboratory of Ophthalmology Zhongshan Ophthalmic Center Sun Yat‐sen University Guangdong Provincial Key Laboratory of Ophthalmology and Visual Science Guangzhou China; ^2^ Center for Orthopedic Surgery The Third Affiliated Hospital of Southern Medical University Guangzhou China; ^3^ Institute of experimental center Guangdong Cord Blood Bank Guangzhou China

Dear Editor,

Treatment for optic nerve (ON) injury remains a challenge worldwide. As essential paracrine signals of mesenchymal stem cells (MSCs), exosomes (exos) show source cell‐like biological functions, which have recently been suggested to promote the functional recovery of central neurons.^1^, ^2^, ^3^, ^4^ Reportedly, MSC‐exos transfer active components, especially microRNAs (miRNAs), to facilitate intercellular communication, thereby exerting tissue regenerative effects.^5^, ^6^, ^7^, ^8^ However, the effect of exos derived from umbilical cord MSCs (HuMSC‐exos) and the underlying mechanisms in ON injury remain unclear. Here, we intended to explore whether HuMSC‐exos have a neuroregenerative effect and the molecular mechanisms by which exosomal miRNAs play a role in this process.

Exos were isolated from HuMSCs (Figure S1A–C) and purified by ultracentrifugation. Purified HuMSC‐exos were identified by positive expression of their specific surface markers (CD9, CD63 and TSG101) by Western blotting (Figure S2A–E). They displayed typical exosomal features, including morphology and size, as detected by transmission electron microscopy (Figure S2F). Nanoparticle tracking analysis revealed that purified HuMSC‐exos were relatively homogeneous particles, and the particle concentration in the distribution range of 30–200 nm was approximately 93.3% (Figure S2G).

In the ON crush (ONC) model, HuMSC‐exos promoted the survival of 30% of the retinal ganglion cells (RGCs) (Figure 1A,B), rescued 60% of the RGCs from apoptosis (Figure 1C,D), increased retinal nerve fibre layer thickness (Figure 1E–G) and promoted axon regeneration (Figure 1H–J) at 21 days after ONC. In addition, pattern electroretinography showed that HuMSC‐exos efficiently recovered the loss of RGC function after ONC (Figure 2A–C). Intravitreal injection of HuMSC‐exos rarely caused retinal detachment, and no other side effects were observed in the ONC model. Therefore, intraocular injection is safe. Consistently, we also observed that 3 × 10^9^ HuMSC‐exos promoted RGC neuritogenesis in retinal cell culture (Figure S3) and were more effective than 3 × 10^9^ BMSC‐exos (Figure 2D–G). In contrast, the higher doses of HuMSC‐exos significantly reduced the number of RGCs with neurites compared with that in the untreated cell culture (Figure 2D,F). Together, these results suggest that a moderate dose of HuMSC‐exos has a neuroregenerative effect after ON injury.

**FIGURE 1:**
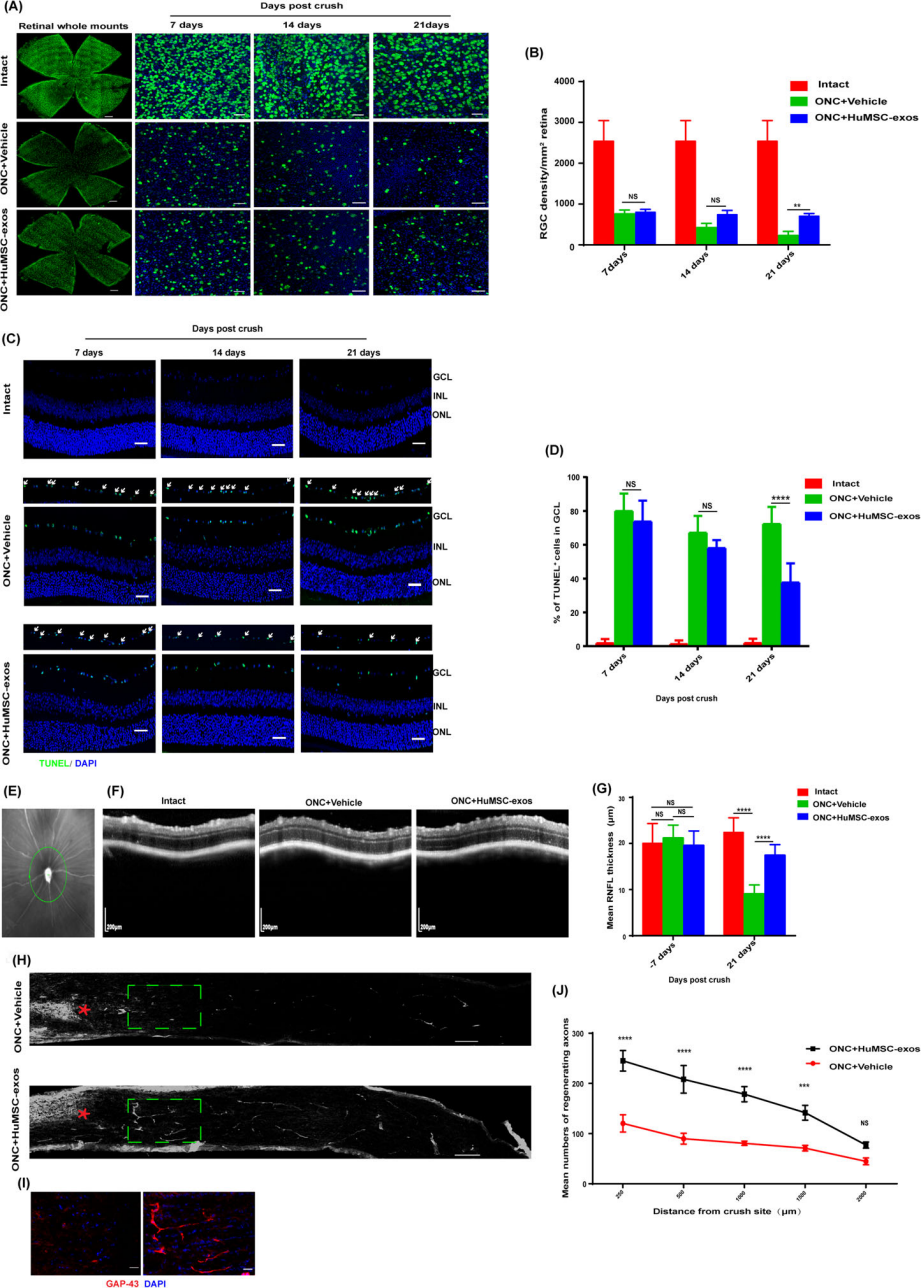
Human umbilical cord mesenchymal stem cells‐derived exosomes (HuMSC‐exos) promote retinal ganglion cell (RGC) survival and axon regeneration in optic nerve injury. (A) Representative confocal images of retinal whole mounts stained with an anti‐Rbpms antibody (green, RGC‐specific marker) and DAPI nuclear staining (blue) in the intact, ONC+HuMSC‐exos and ONC+vehicle groups at 7, 14 and 21 dpc. Scale bar: 50 μm. (B) The average number of RGCs per retina in the intact, ONC+HuMSC‐exos and ONC+vehicle groups. N = eight per group. Data are shown as the mean ± SEM. *p < .05, **p < .01, ns = nonsignificant. (C) RGC apoptosis detection by TUNEL and DAPI staining in mouse retinas of the intact, ONC+HuMSC‐exos and ONC+vehicle groups at 7, 14 and 21 dpc with the labelling of the outer nuclear layer (ONL), inner nuclear layer (INL) and GCL. Scale bar: 50 μm. ONC, optic nerve crush. (D) The mean percentages of RGC apoptosis (%) were statistically analyzed in the ONC+HuMSC‐exo, ONC+vehicle and intact groups at each time point. N = eight per group. Data are shown as the mean ± SEM. *p < .05, ****p < .0001, ns = nonsignificant. (E) RNFL measurements were performed from a section of the retina surrounding the ON head (green line). Scale bars: 200 μm. (F) Representative images of the longitudinal section of the retina from the intact, ONC+vehicle or ONC+HuMSC‐exo groups measured by OCT. Scale bars: 200 μm. (G). Quantification of the mean RNFLT (μm) in the three groups at day 7 prior to ONC and 21 dpc. *N* = eight per group. Data are shown as the mean ± SEM. *p < .05, ****p < .0001, ns = nonsignificant. (H) Representative confocal images showing GAP‐43^+^ regenerating axons (grey) with the vehicle and HuMSC‐exo injections at 21 dpc. Scale bars: 200 μm. (I) The inset shows a higher magnification of the green boxed region in (H). (J) Quantification of the average numbers of regenerating axons for (H) at 250−2000 μm from the crush site. *N* = eight per group. Data are shown as mean ± SEM.*p < .05, **p < .01, ****p < .0001, ns = nonsignificant.

**FIGURE 2:**
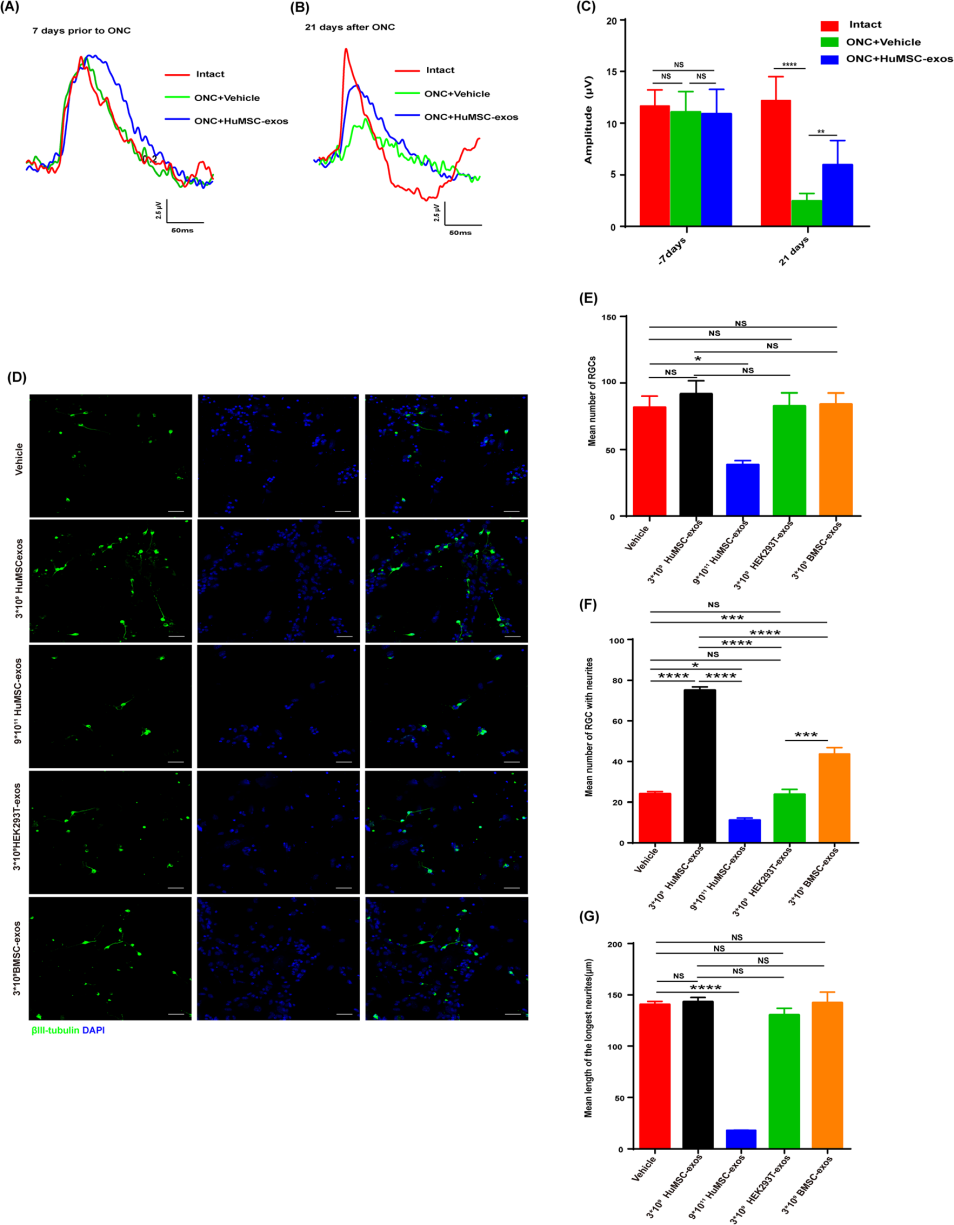
HuMSC‐exos preserve retinal ganglion cell (RGC) functions in vivo and promote RGC neuritogenesis in vitro.(A) Representative traces of observable P waves from the three groups on day 7 prior to ONC to record the P wave baseline. (B) Representative traces of observable P waves from the three groups at 21 dpc. (C) Mean amplitude of P waves measured with PERG from the three groups on day 7 prior to ONC and 21 dpc. *N* = eight per group. Data are shown as the mean ± SEM.*p < .05, **p < .01, ****p < .0001, ns = nonsignificant. (D). Representative confocal images of retinal cells stained with an anti‐βIII‐tubulin antibody (green) and a DAPI (blue) when they were treated with two doses of HuMSC‐exos, HEK293T‐exos, BMSC‐exos and vehicle. Scale bar: 50 μm. (E). Quantification of the mean number of surviving βIII‐tubulin retinal cells from the five groups described in (D). *N* = three per group. Data are shown as the mean ± SEM.*p < .05, **p < .01, ****p < .0001. (F). Quantification of the mean number of βIII‐tubulin retinal cells with neurites from the five groups described in (D). *N* = three per group. Data are shown as the mean ± SEM. *p < .05, **p < .01, ****p < .0001, ns = nonsignificant. (G). The mean length of the longest βIII‐tubulin retinal cell neurite (μm) from the five groups described in (D). *N* = three per group. Data are shown as the mean ± SEM. *p < .05, **p < .01, ****p < .0001, ns = nonsignificant.

An increasing number of studies show that exos as nanocarriers are important mediators that facilitate intercellular communication rather than cell‐to‐cell contact. Previous reports have shown that exo‐mediated miRNA delivery is a new method of intracellular communication and plays a key role in central nervous system (CNS) therapy.^5^, ^6^, ^7^ However, studies on the role of HuMSC‐exos and exosomal miRNAs in ON injury are very rare. Thus, using high‐throughput sRNA sequencing, we analyzed miRNA profiles in HuMSC‐exos. The data showed that 24 specific miRNAs were abundant in HuMSC‐exos compared with HEK293T‐exos (Figure 3A and File S1). KEGG pathway analysis showed that the target genes of these twenty‐four miRNAs were significantly enriched in the mTOR pathway (Figure S4A and File S2). GO functional annotation analysis revealed that the target genes associated with axonogenesis, axon extension and neurogenesis were significantly enriched (Figure S4B and File S3). Next, we verified that eight of 24 miRNAs could be delivered into RGCs by HuMSC‐exos, miR‐22‐3p, miR‐222‐3p, miR‐221‐3p, miR‐21‐5p, miR‐543, miR‐29a‐3p, miR‐24‐3p and miR‐27a‐3p, which accounted for 17.79% in HuMSC‐exos but only 6.65% in HEK293T‐exos (Figure 3B,C). Then, we used TargetScan, miRDB, miRWalk and miRTarBase to identify that these miRNAs all directly target mTOR pathway‐related genes (Figure S4C and File S4).

**FIGURE 3:**
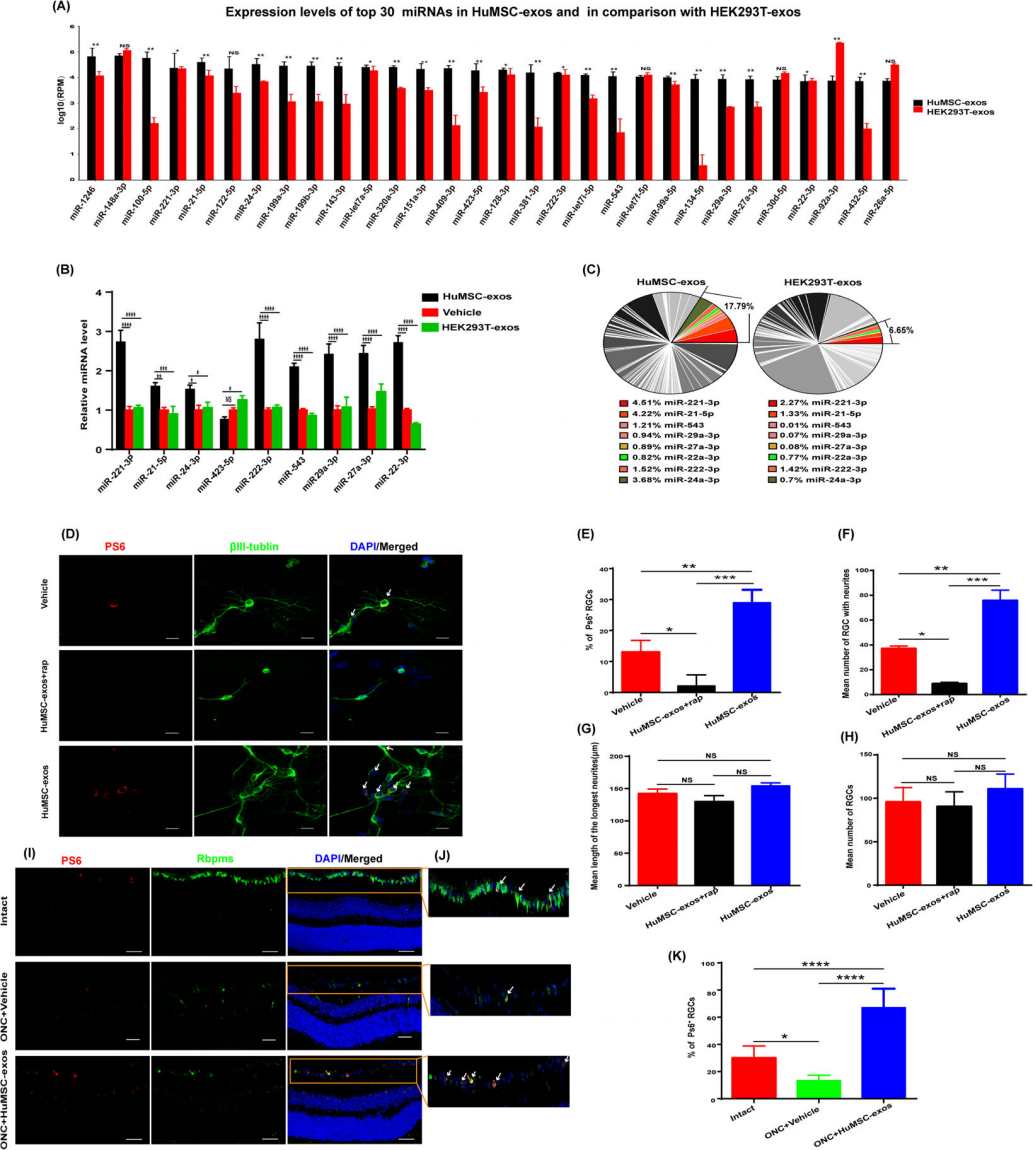
HuMSC‐exos may promote axon regeneration/neuritogenesis through miRNA activation of mTORC1.(A) The high‐throughput small RNA sequencing analysis for the expression levels of top 30 miRNAs in HuMSC‐exos and in comparison with HEK293T. (B) RT‒qPCR analysis showing the relative expression levels of these nine miRNAs in retinal cell cultures treated with HuMSC‐exos, HEK293T‐exos and vehicle served as a control. *N* = three per group. Data are presented as the mean ± SD. *p < .05, **p < .01, ****p < .0001, ns = nonsignificant. (C) Pie chart showing that 17.79% of the 8 miRNAs were verified by RT‒qPCR in HuMSC‐exos, while only 6.65% were verified in HEK293T‐exos via high‐throughput small RNA sequencing. The proportions of the eight miRNAs are labelled by colour. (D) Retinal cells immunostained for βIII‐tubulin (green) and pS6 (red) in cultures, which were incubated with HuMSC‐exos, HuMSC‐exo+rap or vehicle. PS6^+^ RGCs (arrows). rapamycin, rap. Scale bar: 20 μm. (E) Quantification of the proportion of pS6^+^ and βIII‐tubulin^+^ cells among total βIII‐tubulin ^+^ cells in retinal cell cultures from the three groups described in (D). *N* = three per group. Data are presented as the mean ± SEM. *p < .05, **p < .01, ****p < .0001, ns = nonsignificant. (F) Quantification of the mean number of βIII‐tubulin^+^ RGCs with neurites from the three groups described in (D). *N* = three per group. Data are shown as the mean ± SEM. *p < .05, **p < .01, ****p < .0001, ns = nonsignificant. (G) Quantification of the mean length of the longest neurite of βIII‐tubulin^+^ RGCs (μm) from the three groups described in (D). *N* = three per group. Data are shown as the mean ± SEM. *p < 0.05, **p < 0.01, ****p < 0.0001, ns = nonsignificant. (H). Quantification of the mean number of surviving βIII‐tubulin retinal cells from the three groups described in (D). *N* = three per group. Data are shown as the mean ± SEM. *p < 0.05, **p < 0.01, ns = nonsignificant. (I) Images of retinal sections (21 dpc) from the intact (upper), ONC+vehicle (middle) and ONC+HuMSC‐exo (low) groups (left panel) showed that the GCL was immunostained with pS6 (red) and Rbpms (green). Scale bar: 50 μm. (J) Higher magnification sections of the GCL in (I) show pS6+ cells colocalized with Rbpms+ cells, demonstrating pS6+ RGCs (arrows, right panel). (K) Quantification of the proportion of pS6^+^ and Rbpms^+^ cells among total Rbpms^+^ cells in retinal sections from the three groups described in (I). *N* = six per group. Data are presented as the mean ± SEM. *p < 0.05, **p < 0.01, ****p < 0.0001, ns = nonsignificant.

mTOR acts throughtwo signalling complexes called mTORC1 and mTORC2. Activation of mTORC1 leads to the downstream phosphorylation of S6 ribosomal protein (pS6) to initiate protein translation.^9^ Recent studies have shown that an increase in mTORC1 activity may be an important factor for regeneration in the mature CNS.^10^ Therefore, the determination of whether HuMSC‐exos play a regenerative role by regulating mTORC1 activity in ON injury is very important. Immunostaining of pS6 was used to monitor mTORC1 activity, and the results indicated that HuMSC‐exos significantly increased the mTORC1 activity of RGCs both in vitro and in vivo (Figure 3D,E,I,K). Nevertheless, inhibition of mTORC1 activity by rapamycin significantly prevented the neuritogenesis of RGCs (Figure 3D–F), suggesting that the regenerative effects of HuMSC‐exos were mTORC1 activity‐dependent.

To further clarify which exosomal miRNAs delivered into RGCs are truly functional by regulating mTORC1 activity, we selected miR‐222‐3p and miR‐22‐3p, which differed significantly between HuMSC‐exos and HEK293T cells (Figure 3B), to transfect HuMSC‐exos for the subsequent treatment of retinal cells. Next, miR‐222‐3p and miR‐22‐3p mimics (RiboBio, Guangzhou) were transfected into HuMSC‐exos using a transfection kit (EXET10A‐1, SBI), and then, retinal cells were treated with these exos (Figure 4A,B). The quantitative real‐time PCR (RT‒PCR) data verified that miR‐222‐3p and miR‐22‐3p expression in retinal cells was upregulated (Figure 4C). Moreover, the mTORC1 activity of RGCs (Figure 4D,E) and the number of RGCs with neurites (Figure 4D,F ) were obviously increased by HuMSC‐exos transfected with these two miRNA mimics. HuMSC‐exos transfected with miR‐222‐3p and miR‐22‐3p inhibitors had the opposite effect compared to that of the inhibitor NC group (Figure 4D–F). These results demonstrate that miR‐222‐3p and miR‐22‐3p derived from HuMSC‐exos play a crucial role in promoting axon regeneration by activating mTORC1 activity.

**FIGURE 4:**
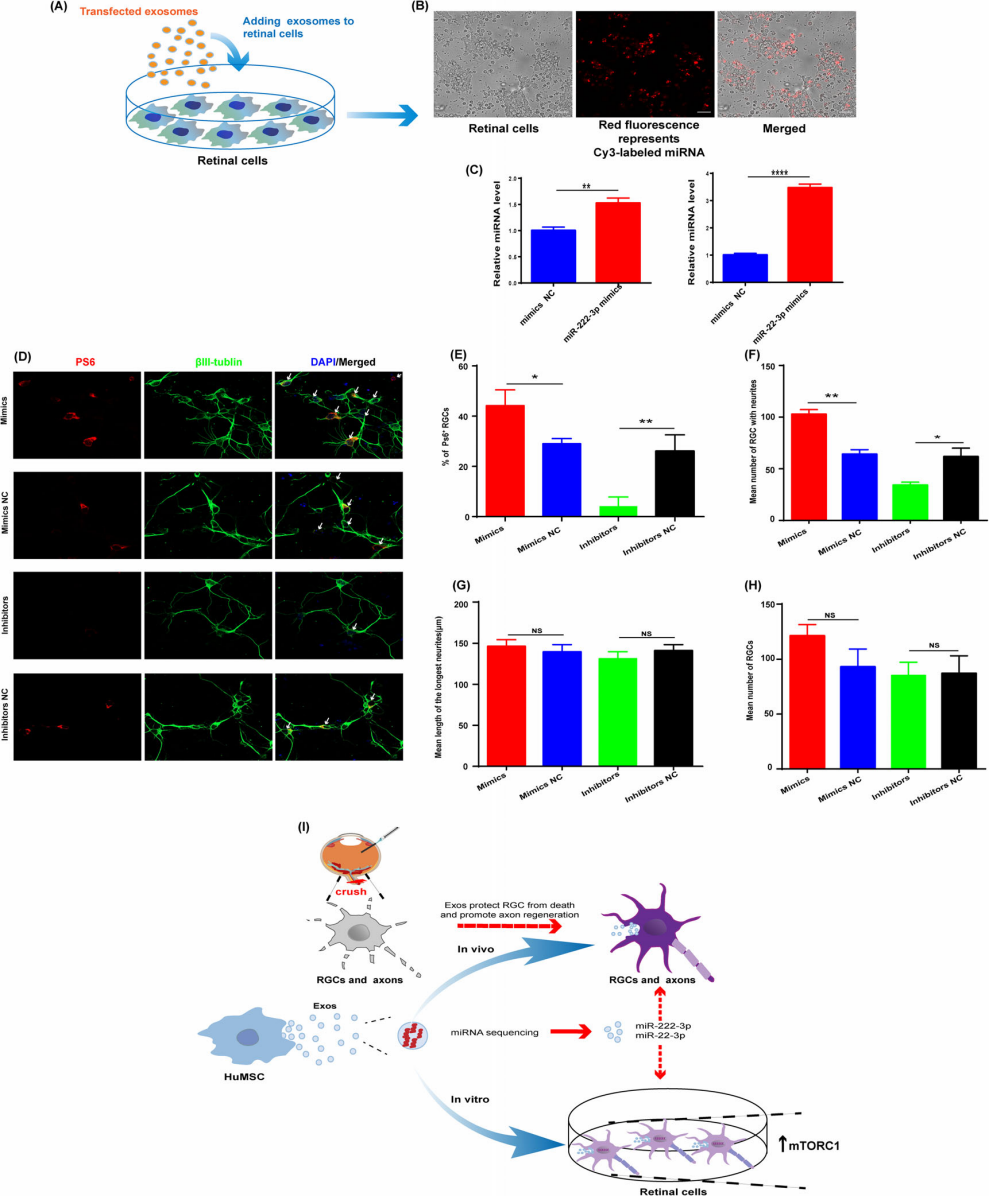
HuMSC‐exos promote neuritogenesis through miR‐222‐3p and miR‐22‐3p‐dependent mTORC1 signaling. (A) HuMSC‐exos transfected with Cy3‐labeled NC were added to target retinal cells. (B) The image of retinal cells indicates that the Cy3‐labeled miRNA cargoes were successfully delivered. Scale bar: 20 μm. (C) RT‒qPCR was conducted to evaluate the levels of miR‐222‐3p and miR‐22‐3p. N = three per group. Data are presented as the mean ± SD. *p < .05, **p < .01, ****p < .0001. (D) Retinal cells immunostained for βIII‐tubulin (green) and pS6 (red) in cultures, which were treated with HuMSC‐exos transfected with miR‐222‐3p and miR‐22‐3p mimics, inhibitors, mimics NC and inhibitors NC. PS6^+^ retinal ganglion cells (RGCs) (arrows). NC, negative control. Scale bar: 20 μm. (E) Quantification of the proportion of pS6^+^ and βIII‐tubulin^+^ cells among total βIII‐tubulin ^+^ cells in retinal cell cultures from the above four groups described in (D) *N* = four per group. Data are presented as the mean ± SEM.*p < .05, **p < .01. ns = nonsignificant. (F) Quantification of the mean number of βIII‐tubulin^+^ RGC with neurites from the four groups described in (D). *N* = four per group. Data are shown as mean ± SEM. *p < .05, **p < .01, ns = nonsignificant. (G) Quantification of the mean length of the longest neurite of βIII‐tubulin^+^ RGCs(μm) from four groups described in (D). *N* = four per group. Data are shown as the mean ± SEM. *p < .05, **p < .01, ns = nonsignificant. (H) Quantification of the mean number of surviving βIII‐tubulin retinal cells from the four groups described in (D). *N* = four per group. Data are shown as the mean ± SEM.*p < .05, **p < .01, ns = nonsignificant. (I) Schematic representation of HuMSC‐exos promoting axon regeneration during ON injury. In the optic nerve crush (ONC) model, HuMSC‐exos protect RGCs from death and promote axon regeneration, which may depend on exosomal miR‐222‐3p and miR‐22‐3p to activate mTORC1 signalling.

In summary, our study shows that HuMSC‐exos are a promising and viable regenerative therapy for ON injury that depends on specific miRNAs to regulate mTORC1 signalling. We also provide a better understanding of the important role of miR‐222‐3p and miR‐22‐3p delivered by HuMSC‐exos in promoting axon regeneration. The schematic representations of our study are shown in Figure 4I and Figure S2H. Therefore, these results may provide new evidence for the role of HuMSC‐exos and their exosomal miRNAs in axon regeneration after ON injury. Moreover, modified HuMSC‐exos can be further transfected with appropriate miRNAs to augment their efficacy in neuroregenerative therapy.

## CONFLICT OF INTEREST STATEMENT

The authors declare no conflict of interest.

## Supporting information



Supporting Information

Supporting Information

Supporting Information

Supporting Information

Supporting Information

Supporting Information

Supporting Information

Supporting Information
