# Supplementary figures and images for: Umbilical cord mesenchymal stem cell‐derived exosomes promote axon regeneration during optic nerve injury through microRNA‐dependent mTORC1 signalling
Source: Clin Transl Med. 2023 Jul 3;13(7):e1319. doi: 10.1002/ctm2.1319 (PMC10318125; doi:10.1002/ctm2.1319)

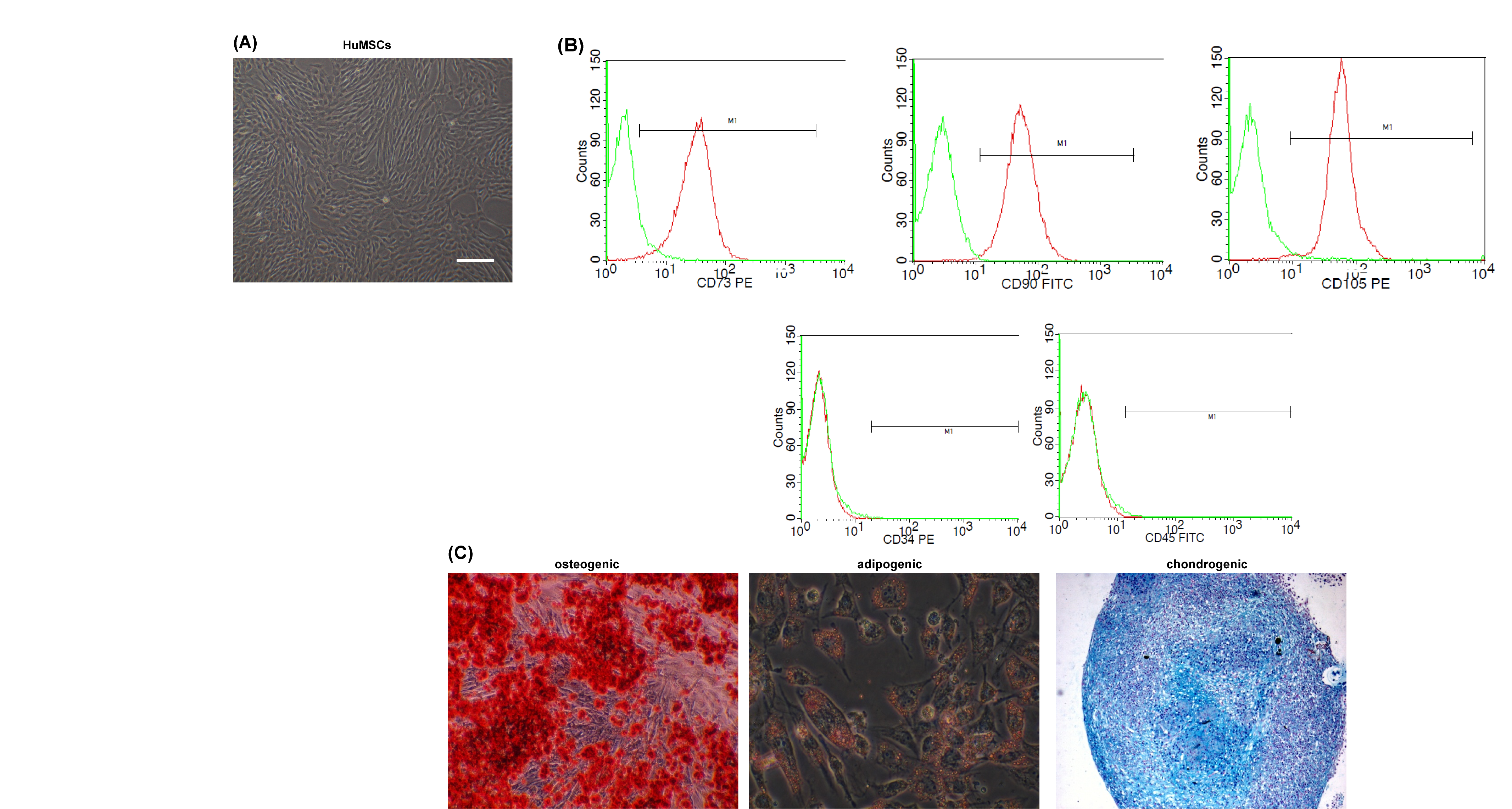

Supplement: Supplementary file 5 — Supporting Information [file CTM2-13-e1319-s002.tif]

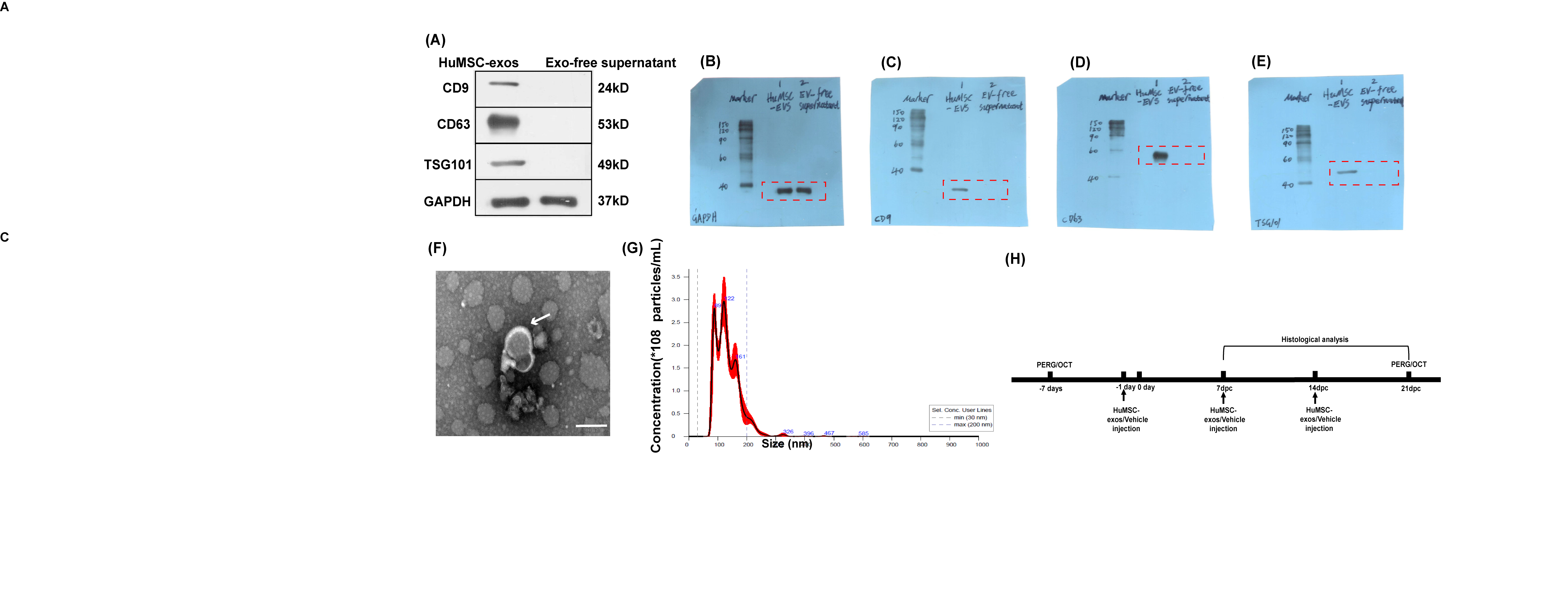

Supplement: Supplementary file 6 — Supporting Information [file CTM2-13-e1319-s004.tif]

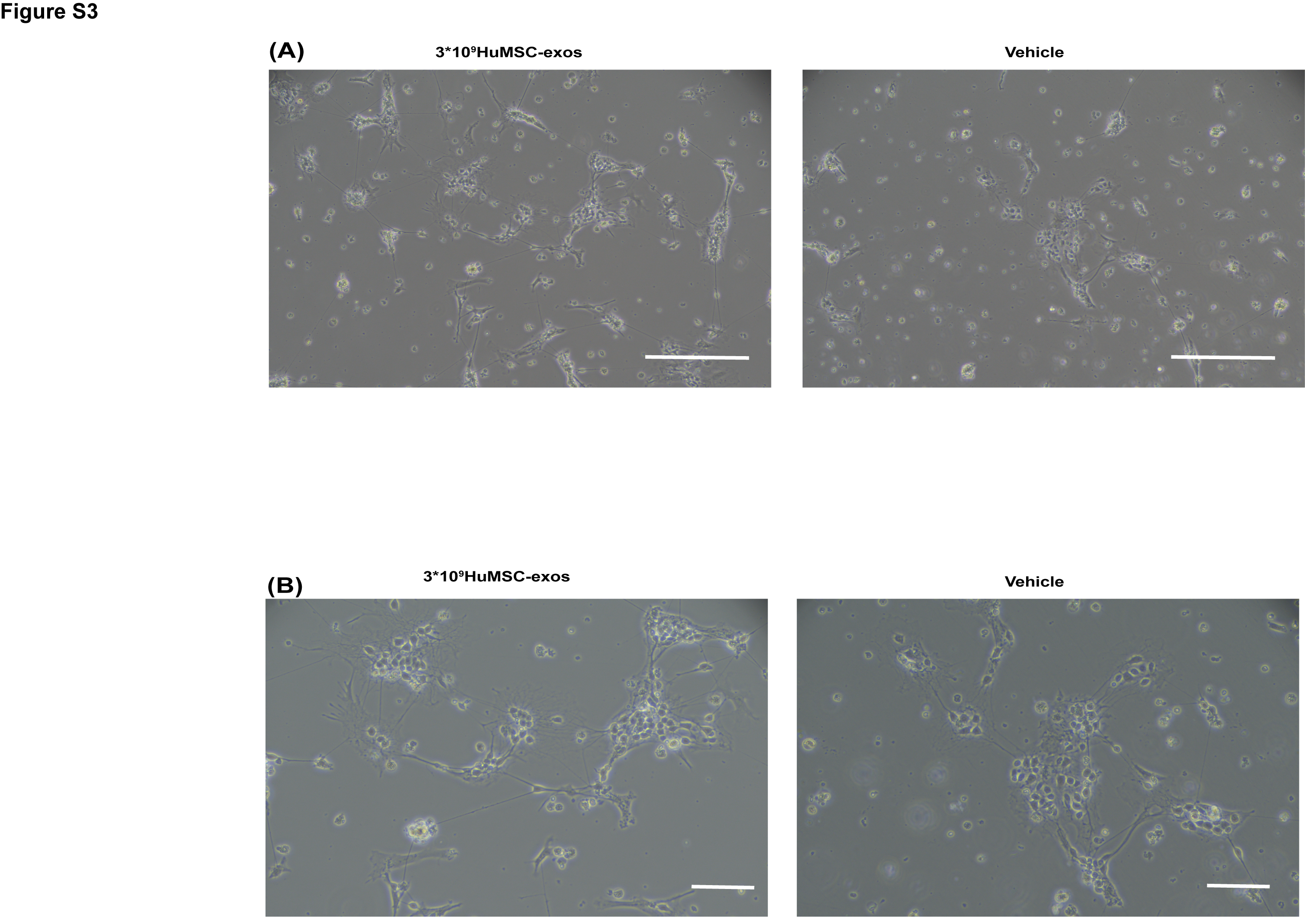

Supplement: Supplementary file 7 — Supporting Information [file CTM2-13-e1319-s007.tif]

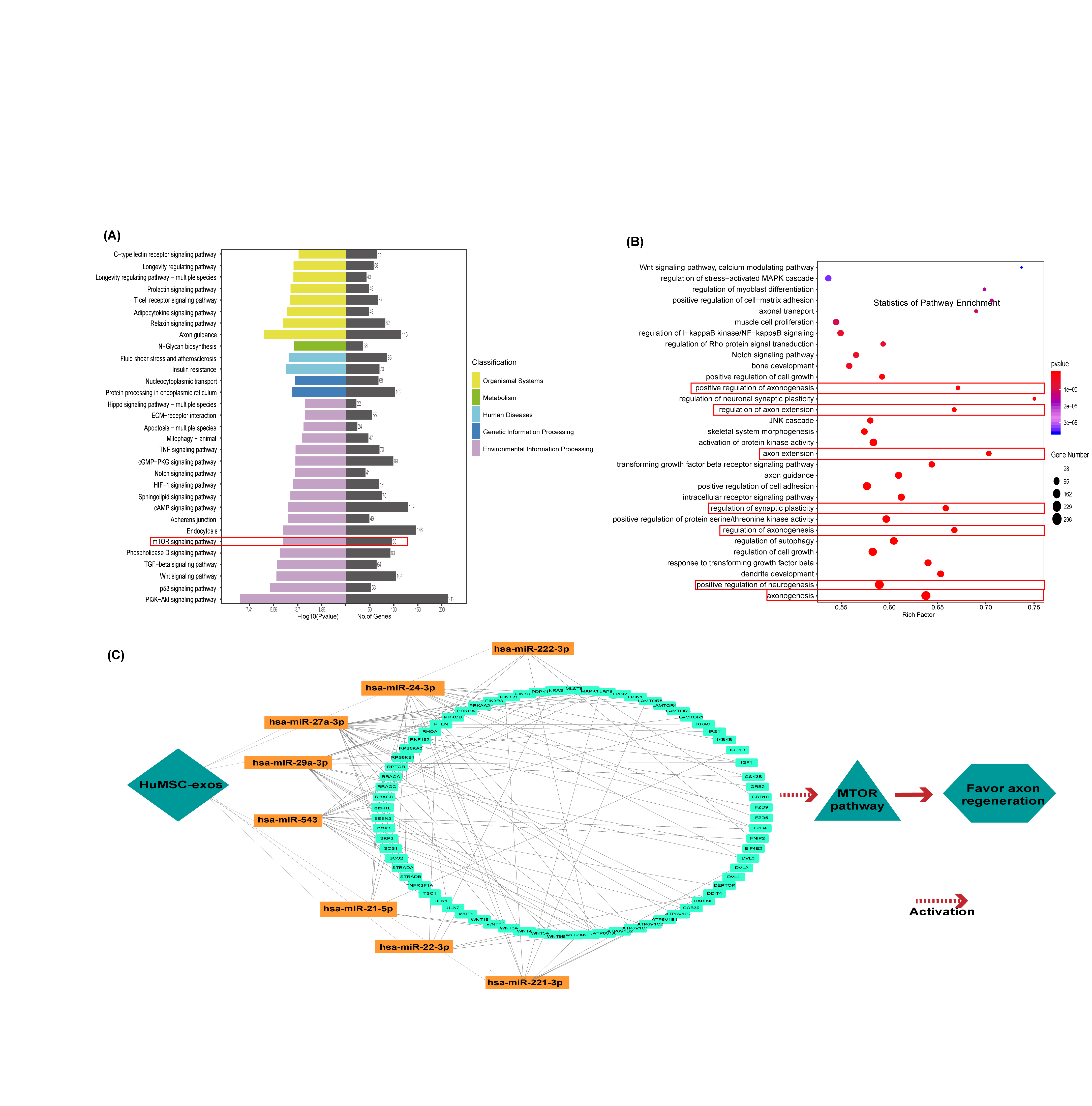

Supplement: Supplementary file 8 — Supporting Information [file CTM2-13-e1319-s001.tif]
